# The Role of Personal Goals in Depressive Reaction to Adverse Life Events: A Cross-Sectional Study

**DOI:** 10.1100/2012/810341

**Published:** 2012-12-05

**Authors:** Alessandro Couyoumdjian, Cristina Ottaviani, Roberta Trincas, Grazia Spitoni, Katia Tenore, Francesco Mancini

**Affiliations:** ^1^Department of Psychology, Sapienza University of Rome, Via dei Marsi 78, 00185 Rome, Italy; ^2^Department of Psychology, University of Cagliari, Via Is Mirrionis 1, 09123 Cagliari, Italy; ^3^IRCCS, Santa Lucia Foundation, Via Ardeatina 306, 00142 Rome, Italy; ^4^Scuola di Psicoterapia Cognitiva S.r.l., Viale Castro Pretorio 116, 00185 Roma, Italy

## Abstract

Consistent with cognitive views of depression, we aimed to investigate the mediating role of personal goals in the relationship between stressful events and distinct patterns of depressive symptoms in a nonclinical sample. Participants identified a dysphoric episode that occurred in the previous year by reporting the severity of 12 depressive symptoms and their plausible cause. A goal taxonomy was used to determine how much the event interfered with the achievement of a series of personal goals. After controlling for age and current level of depression, the patterns of symptoms differed based on the triggering events. The relationship between sadness and affective losses was partially mediated by the personal goal of lovableness, and success was a partial mediator in the association between an event of failure and symptoms of worthlessness and anhedonia. Although the cross-sectional design of the study does not allow for conclusions on the direction of effects, findings suggest the importance of motivational factors in the development of specific patterns of depressive symptoms to adverse events. Assuming a continuum from low mood to clinical depression, treatment models could benefit from a precise identification of the specific stressors that initiate depressive behaviour and the personal meaning assigned to those events.

## 1. Introduction

Life stress is one of the main precipitating factors of depression with the consequence of overdiagnosis and hyper-prescription of antidepressants beyond their true utility [1]. A dimensional perspective of psychopathology assumes the existence of a continuum from low mood to clinical depression, implying that depressive disorders could be understood by investigating normal depressive reactions [2]. Firmly grounded in these basic assumptions are evolutionary theories, according to which depressive symptoms are selected as a consequence of environmental pressure [3]. An integrative framework for understanding the evolutionary origin of depression has been introduced and tested by Keller and colleagues [4–6] under the name of Situation-Symptom Congruence Hypothesis. This Hypothesis postulates that specific kinds of adverse life events (ALEs) evoke different dysphoric episodes that increase the ability to adaptively cope with the challenges specific to each situation. The Hypothesis has been empirically supported showing that on one hand that social losses (death of a loved one, romantic breakups, and social isolation) were associated with more emotional pain, crying, desire for social support, and appetite loss. On the other hand, failure to reach a goal, chronic stress, and winter seasons were associated with more guilt, hopelessness, fatigue, and pessimism [5]. The authors replicated the patterns in clinically depressed people [6]. These results have important clinical implications, as they suggest that the depressive syndrome is not a unitary phenomenon.

Despite its advantages, this Hypothesis is not without limits, in fact it ignores that ALEs are not necessarily stressful to all people [7] and may also have positive effects. People may, for example, perceive positive changes in self-concept after the occurrence of ALEs, such as feeling a stronger person, more mature, and better able to cope with other crises [8]. Indeed, categories of ALEs can be taken into account only if we implicitly assume that these events have a shared meaning among people (i.e., a prototypical appraisal). For most people, the death of a family member represents a severe affective loss but in the case of ambivalence towards the person, this event might assume a different meaning. Moreover, a prototypically positive life event, such as childbirth, may lead to severe depressive symptoms (i.e., postpartum depression). Apparent insignificant changes or minor events in a person's life may cause a depressive reaction because of the meaning attributed to them.

Although much research has demonstrated a link between ALEs and depressive symptoms, relatively little research examined the cognitive mechanisms that may occur in this relationship. Competence-related factors such as self-efficacy, self-esteem, self-confidence, and negative attributional style have often been proposed as potential moderators but empirical tests yielded inconsistent results [9, 10]. An exception is provided by Kopala-Sibley and Zuroff [11], who showed that self-critical and dependent moods were significant mediators of the association between depressive symptoms and threats to self-worth and to relationships, respectively.

According to a cognitive perspective, emotions derive from the subjective interpretations of the triggering events and such appraisal reflects individual's goals investment [12]. Clinical findings suggest that depressed people tend to set excessively high goals [13] or often adopt self-worth goals, seeking to prove self-worth and to avoid proof of worthlessness [14]. Moreover, depressed individuals typically overinvest in one goal, but underinvest in other areas of their lives [15], as confirmed by a longitudinal study [16]. 

Two of the most clear diathesis-stress models of depression [17] have been developed in a similar way by Beck [18] and Blatt [19]. According to them, individual differences in the cognitive and psychodynamic constructs of sociotropy and dependence, or autonomy and self-criticism are viewed as responsible for depressive vulnerability. In other terms, the value attributed to sociality and achievement determines how stressors will be interpreted, leading or not to depression. A person with high sociotropic values would be more likely than someone without such beliefs to interpret an interpersonal loss experience as highly significant, thus potentially triggering depressive symptoms. Conversely, someone with high autonomy is more likely to develop a depressive reaction after an event of personal failure.

Our main objective was to investigate the relevance of personal goals in the situation-symptom relationships. Based on Beck and Blatt conceptualizations, we hypothesized that (1) the relationships between affective losses and symptoms of sadness and crying would primarily exist when the individual believes that the event interfered with personal goals such as lovableness; (2) the relationships between events of failure and symptoms of worthlessness and anhedonia would exist primarily when the individual evaluates the event as preventing the achievement of goals focused on personal success.

To our knowledge, Keller et al.'s hypothesis was a first attempt to go over the simplistic view that depression exists as consequence of a single evolutionary function. In a similar vein, we investigated how different personal goals are involved in specific depressive symptoms, with the aim to understand the psychological factors involved in low mood reactions to stressors. At the same time, our study aspires to improve cognitive theories of depression, mainly focused on the identification of general dysfunctional appraisals.

## 2. Methods

### 2.1. Participants

The study was conducted through an online survey and participants were recruited via several professional mailing lists. Seven hundred and eighty-nine participants agreed to participate and 511 completed the study. In all, 456 reported the occurrence of a 5-day period of low mood during the previous 12 months. The final sample was composed of 328 women (mean age = 38.8.1 (10.5) years) and 128 men (mean age = 42.7 (9.7) years). All subjects were Caucasians. The majority of the participants were in paid employment (*n* = 397) and had completed a higher vocational or University education (*n* = 364).

### 2.2. Procedure

The survey was administered in a single session by http://Questionpro.com, which guarantees the privacy and confidentiality of the respondents, and it took about 45 min to complete. As the online administration provided us with a measure of the time taken to fill out the questionnaire, we were able to exclude outliers (less than 2% of the respondents). After providing instructions and informed consent, all respondents completed a series of forms in the same order as presented below. After survey completion, participants were debriefed and thanked for their time. Debriefing consisted in a brief explanation of the background and aim of the study, and a feedback on subjects' personal scores at standardized questionnaires. The study was conducted according to the Declaration of Helsinki guidelines.

### 2.3. Sociodemographic and Personal Information

Participants were asked to complete a sociodemographic form, which included items regarding age, gender, education, ethnicity, and employment.

### 2.4. Mood State during the Previous Week

To assess current depressive state, we administered the Italian version of the Center for Epidemiologic Studies Depression Scale (CES-D; [20]) which showed good reliability and convergent validity with related self-report measures. The CES-D is a 20-item self-report scale that assesses the frequency of occurrence of symptoms of depression during the past week. Total score ranges from 0 to 60. Standard cutoffs are >16 for mild depression and >23 for clinical depression. 

### 2.5. Past Episode of Depressive Symptoms

We included the same questions used by Keller et al. [6] in their interviews. First, we assessed the occurrence of the worst dysphoric episode over the previous year by investigating the presence of depressive symptoms for at least 5 days, a period considered adequate by the authors. Such symptoms represented the disaggregated nine symptoms of criterion A for major depression in DSM-IV (sadness, anhedonia, fatigue, psychomotor retardation, restlessness, insomnia, hypersomnia, appetite loss, appetite gain, self-harm, poor concentration, worthlessness). Participants were asked to signal how much each symptom interfered with their daily life (from 0, absence of the symptom, to 4, symptom interfering completely with daily life). If they did not experience a dysphoric episode in the previous year (i.e., they scored 0 to all symptoms), they automatically exited the survey. Then, we asked if something happened that might have contributed to make them feel that way, and possible answers were: (a) no, I cannot identify any particular event and (b) yes, the symptoms are the consequences of particular events. When the answer was negative, participants were associated with the “no specific cause” category; when it was affirmative, they were asked to further identify the plausible reason for this period of low mood (ALE), first describing it in a free-format paragraph, and then selecting the single most likely cause from Keller et al.'s [6] categories (failure, health problems, interpersonal conflict, death of a loved one, romantic breakup, stress, scare, and other).

### 2.6. Personal Goals

Participants successively reported the degree to which the recognized ALE interfered (from 0, not at all, to 4, totally) with the achievement of a series of personal goals (e.g., “having a job that is rewarding, satisfying,” “developing and enhancing my relationship with my spouse”), included in the 29 clusters (e.g., occupation, marriage) of the Italian adaption of a hierarchical goal taxonomy [21]. The taxonomy showed high inter-item consistency for each cluster (standardized Cronbach's alphas from .89 to .61) and high levels of replicability across different age groups.

### 2.7. Statistical Analysis

Data analyses were performed with SPSS 18. Level of significance was set at *P* < .05. In case of significant effects, Fisher LSD post hoc tests were carried out. Three experimenters independently coded each free-format description of the events into one of the ALE categories. Then, these categories were compared with those indicated by the subjects. When at least two raters agreed with the participant (77.8% of the time), that category was used. If this was not the case, the majority category was used instead of the participant's (22.1% of the time). Due to the small Ns, the precipitants “scare” and “other” were either recategorized, if possible, or excluded from the analyses. Standardized symptom scores were obtained for each participant. Differences in symptoms and goals interference due to gender were analyzed by *t*-test. Pearson correlations were computed between all the variables of interest.

#### 2.7.1. Preliminary Analyses: Replication of the Situation-Symptom Hypothesis

As our main hypothesis relies on the existence of a relationship between specific stressful events and different depressive reactions, the preliminary aim of our study was to replicate Keller et al. [6]. The prediction that specific ALE categories were associated with different depressive symptom patterns was first tested by the “event X symptom” interaction term in a repeated-measures multivariate analysis of variance (MANOVA). The standardized depressive symptoms served as repeated measures dependent variables and the event categories served as between-subjects predictor variables. To control for current mood [6], CES-D was used as a covariate.

#### 2.7.2. Main Analyses: Testing the Situation-Goals-Symptom Hypothesis

To test for the relationships between appraisal, situation, and symptoms, a series of meditational analyses were performed. Mediation exists when a predictor (*X*, life event) affects a dependent variable (*Y*, depressive symptom) indirectly through at least one intervening variable, or mediator (*M*, goal interference). The Preacher and Hayes SPSS Macro for Multiple Mediators [22] was used to estimate path coefficients and to generate both bootstrap confidence intervals and Normal Theory Tests for total and specific indirect effects of *X* on *Y* through *M*. The number of bootstrap resampling was set at 20.000.

Potential mediators were selected on the basis of theory. Based on our hypotheses, goals [21] related to “Success” and “Lovableness” were preselected and principal component analysis was performed on this subsample (rotation: Varimax, extraction method: eigenvalue > 1). The factors extracted were used as mediators. Dependent variables were the symptoms that resulted significant at a second omnibus MANOVA, run leaving out the “no specific cause” category for the reason that it could not interfere with the achievement of any personal goal. Predictors were the events of failure and affective loss (death of a loved one or romantic breakup).

## 3. Results

The final categories distributions were: failure (*n* = 42), health problems (*n* = 40), interpersonal conflict (*n* = 69), death of a loved one (*n* = 27), romantic break-up (*n* = 63), stress (*n* = 115), and no specific cause (*n* = 100).

*t*-tests results showed no differences in symptoms or goal interference due to gender. Time from the event and symptoms were not correlated. As age was significantly related to some symptoms, this variable was used as a covariate in subsequent analyses.  Table 1 shows means, standard deviations, and intercorrelations for the variables of interest.

### 3.1. Results on the Situation-Symptom Hypothesis

**Figure 1:**
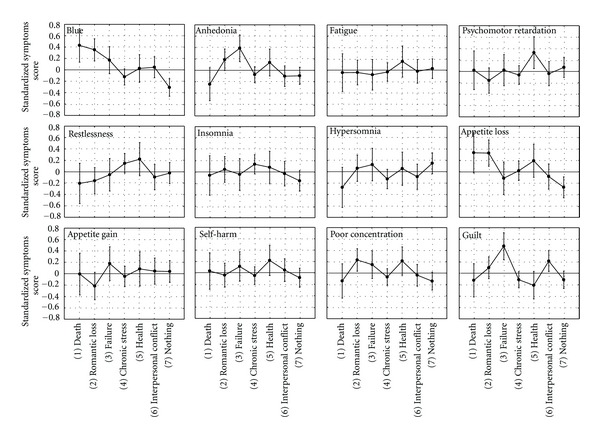
Profiles of average depressive symptom levels for the seven ALEs categories.

The event-by-symptom MANOVA interaction term was significant for the 12 depressive symptoms, *F*(66,4917) = 2.41, *P* < .0001; *η* ^2^ = .04. Symptom levels differed significantly between the event categories for 5 of the 12 symptoms: sadness (*F*(6,447) = 6.93, *P* < .0001), appetite loss (*F*(6,447) = 3.76, *P* = .001), worthlessness (*F*(6,447) = 4.93, *P* < .0001), poor concentration (*F*(6,447) = 2.34, *P* = .03), and anhedonia (*F*(6,447) = 3.66, *P* = .001) (see Figure 1).

**Figure 2:**
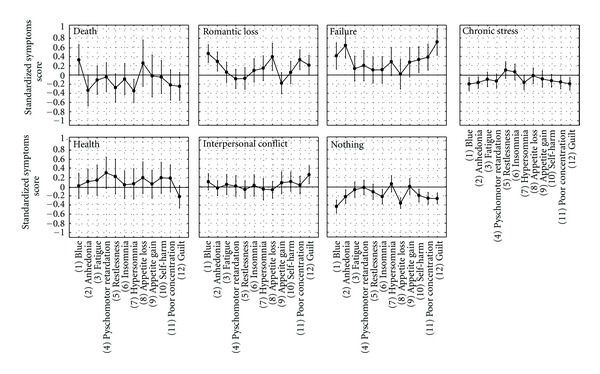
Profiles of average depressive symptom levels for the seven ALEs categories.

Another way to look at SSC is to run a series of one-way ANOVAs on symptoms levels for each event (see Figure 2). Results showed that the average symptom level was different for reactions with no precipitating cause, *F*(11,1067) = 2.62; *P* < .0001), and stress (*F*(11,1232) = 3.91; *P* < .0001), while interpersonal conflict, health problems, and failure showed a trend toward significance (*P* = .07). Post hoc comparisons indicated that reactions that had no precipitating cause were characterized by higher levels of hypersomnia and appetite gain and lower levels of feeling blue and appetite loss compared to the other symptoms (*P* < .05), while stress was associated with higher levels of restlessness and insomnia (*P* < .05) compared to all the other symptoms except for appetite loss.

### 3.2. Results on the Situation-Goals-Symptom Hypothesis

Following principal component analysis, two factors, labelled “lovableness” (sex and romance, marriage, and family) and “Success” (belonging, social recognition and approval, positive social qualities, leadership, social awareness, achievement, personal growth, career, and finances), were used as mediators in the subsequent analyses. Those two factors accounted for 59.2% of total variance.

Results from the event-by-symptom MANOVA yielded a significant interaction effect across the 12 depressive symptoms (*F*(55,3828) = 2.01, *P* < .0001; *η* ^2^ = .03). Significant symptoms, subsequently used as dependent variables, were sadness (*F*(5, 348) = 4.23, *P* = .0009), worthlessness (*F*(5,348) = 5.01, *P* = .0002), and anhedonia (*F*(5,348) = 3.86, *P* = .002). 

Based on our hypotheses, the first model aimed to test the mediating effect of personal goals “lovableness” (*M1*) and “success” (*M2*) in the relationship between an event of affective loss (*X*) and the symptom of sadness (*Y*). The second and third models tested the mediating effect of the same personal goals in the relationship between an event of failure (*X*) and symptoms of worthlessness (*Y*) and anhedonia, respectively (*Y*). Requirements for meditational analysis were met by the intercorrelations between predictors, mediators, and dependent variables, except for the absence of correlations between “lovableness” (*M1*) and failure (*X*) and between “success” (*M2*) and affective loss (*X*) (*P* = .74 and *P* = .12, resp.). For this reason, the use of the second mediator was excluded from the models.

**Figure 3:**
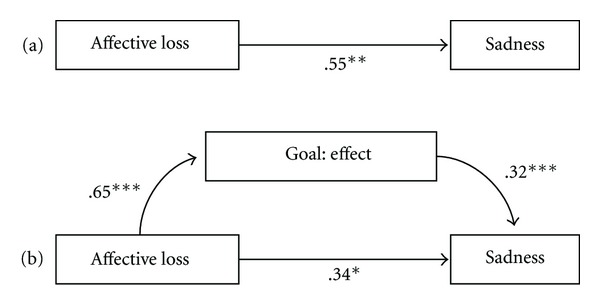
Path models showing total effect (a) and mediated effect (b) of affective loss on sadness. Note: coefficients are unstandardized. *P < .05; **P < .001; ***P < .0001.

Figure 3 shows results for the first mediation model. Events of affective loss (*X*) predicted the occurrence of sadness (*B* = .55, SE = .17, *t* = 3.16, *P* = .002) but this relationship became less significant when the goal of “lovableness” was included in the equation (*B* = .34, SE = .17, *t* = 1.96, *P* = .05). As expected, “lovableness” was a partial mediator of the relationship between affective loss and sadness (indirect effect = .21, SE = .06, 95% CI = .10, .36), as further confirmed by Sobel test (*z* = 3.42, *P* = .001).

**Figure 4:**
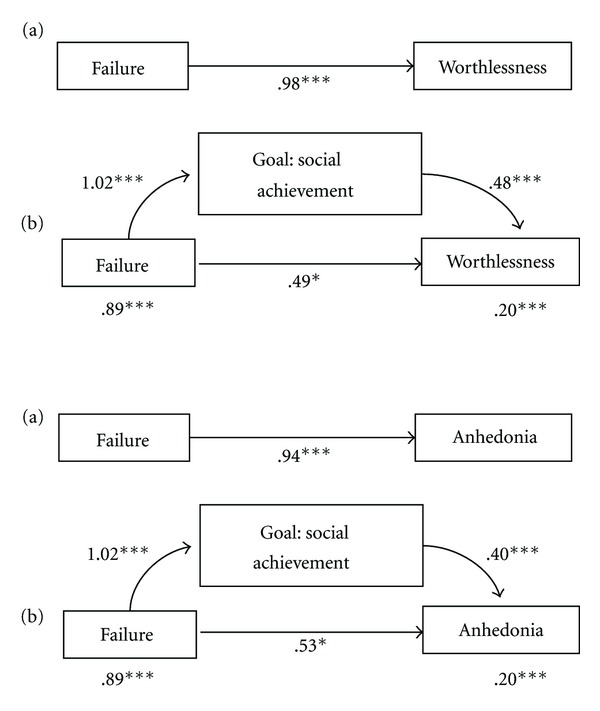
Path models showing total effect (a) and mediated effect (b) of failure on worthlessness (upper section) and anhedonia (lower section). Note: coefficients are unstandardized. *P < .05; **P < .001; ***P < .0001.

In the second and third models, events of failure predicted the occurrence of worthlessness (*B* = .98, SE = .22, *t* = 4.51, *P* < .0001) and anhedonia (*B* = .94, SE = .23, *t* = 4.10, *P* = .0001) but these relationships became less significant when “success” was included in the equations (*B* = 49, SE = .22, *t* = 2.27, *P* = .02, and *B* = .53, SE = .23, *t* = 2.28, *P* = .02, resp.). As expected, “success” partially mediated the relationships between failure and worthlessness (indirect effect = .49, SE = .12, 95% CI = .29, .76; *z* = 4.76, *P* < .0001) (see Figure 4, upper level) and between failure and anhedonia (indirect effect = .41, SE = .11, 95%  CI = .22, .66; *z* = 4.12, *P* < .0001) (see Figure 4, lower level), respectively.

Results did not change when the mediation analyses were conducted with CES-D score as a covariate.

## 4. Discussion

This study explored the relationships between ALEs, depressive symptoms, and appraisals, operationalised as the degree of events interference with personal goal achievement. Findings suggest that the relevance of a specific goal for the individual provides an important source of information that should not be ignored. Goals concerning the domain of lovableness were partial mediators of the relationships between affective losses and sadness, while no link with goals concerning success was observed. Conversely, the associations between an event of failure and symptoms of worthlessness and anhedonia were partially mediated by the importance attributed to success, but not lovableness-related goals. Our exploratory findings suggest that an event has the potential to evoke a specific pattern of symptoms only if it interferes with our life goals. It would be simplistic to associate an event of professional failure with the development of anhedonia, if building a career is not of any importance for the person. This is in agreement with a study on pathological depression conducted by Iacoviello and colleagues [23] in which the interactions between personality types (sociotropy-interpersonal versus autonomy-achievement) and events by themselves were not associated with the number, duration, or the overall chronicity of depressive episodes. Conversely, depressive symptoms were significantly explained by the underlying Independent Goal Attainment factor.

The present study replicated Keller and Nesse's results on the association between different ALEs and specific depressive symptoms. We suggest the possibility to identify (at least) two kinds of depressive reactions associated with two types of ALE. In line with Keller et al. [6], worthlessness and anhedonia were prominent following failed efforts, while sadness was prominent following affective losses. Present findings also provide a plausible explanation for the paradox of depression [24], apparently characterized by patterns of symptoms serving opposite functions: on one hand crying signals the investment in what it is lost; on the other hand, anhedonia suggests the absence of investment in getting it back.

Consistent with Keller and colleagues [6], failure and interpersonal conflict did not evoke different patterns of symptoms. In our sample, however, neither health problems, nor romantic breakup, nor death were characterized by specific patterns of symptoms. The inadequate sample sizes of these precipitants, however, may be a possible reason for the lack of replication.

As in Keller et al.'s [6], reactions that had no recognized reason were prominently characterized by somatic symptoms. This result is particularly interesting if we consider that many patients deemphasize psychosocial symptoms while emphasizing pain as their primary or sole complaint [25]. Moreover, patients with residual physical symptoms following treatment for depression appear to be those at higher risk of relapse. We can speculate that the absence of psychological symptoms, such as sadness and anhedonia, leads these patients to misattribute such reactions to an organic condition, and then makes it more difficult for clinicians to make a correct diagnosis of depression.

Several limitations need to be acknowledged. First, the cross-sectional design of the study precludes any conclusions about the direction of effects. It has to be noted, however, that a recent longitudinal twin study showed that the relationship between ALE and depressive reaction is precisely bidirectional and suggested that reducing life event exposure would reduce depressive symptoms and lowering depressive symptoms would decrease the occurrence of ALE [26]. Also, problems in data interpretation may arise from the use of retrospective self-reports. With regard to reliability issues, a general worsening of memory should increase both inter- and intraindividual variability leading to low or null covariations [27]. Participants' present mood may also have biased their responses. A mood-congruent memory bias has been observed in depressed individuals [28]. For example, in their meta-analysis, Matt and colleagues [29] showed that depressed individuals selectively remember information relevant to their current concerns. Although we cannot exclude the occurrence of this bias in our high CES-D participants, in such a circumstance we would only expect a nonspecific effect. Conversely, our results highlighted specific correlations between events, personal goals, and symptoms that are consistent with our hypotheses. Third, as complete mediation was not found, it can be either assumed that other important mediators remain to be identified or that the taxonomy we used may have not be sensitive enough to detect the multifaceted dimension of people's goals. Fourth, our results cannot be generalized to psychopathological conditions. Finally, as we selected people that experienced a dysphoric episode in the previous year, our sample cannot be considered representative of the general population.

Limitations, notwithstanding, our findings support cognitive models of depression and claim for the need to focus on specific patters of symptoms in order to understand the origins of cognitive vulnerability to depression.

## Figures and Tables

**Table 1:** Means, standard deviations, ranges, and correlations for the variables of interest.

	Mean (SD)	Range	1	2	3	4	5	6	7	8	9	10	11	12	13	14	15	16
(1) Age	39.9 (10.5)	19–66	1.00	−.09	−.11	−.04	−.03	.01	−.13*	.09	−.14*	−.16*	.07	.02	−.02	−.17*	.00	−.04
(2) CES-D	16.1 (11.2)	0–57		1.00	.59*	.63*	.49*	.44*	.34*	.39*	.35*	.29*	.25*	.54*	.60*	.60*	.29*	.37*
(3) Blue	3.1 (1.3)	1–5			1.00	.67*	.46*	.31*	.31*	.41*	.31*	.39*	.17*	.46*	.59*	.61*	.28*	.21*
(4) Anhedonia	2.4 (1.4)	1–5				1.00	.47*	.42*	.46*	.38*	.30*	.35*	.19*	.50*	.66*	.64*	.25*	.32*
(5) Fatigue	2.7 (1.2)	1–5					1.00	.57*	.38*	.29*	.40*	.21*	.28*	.31*	.52*	.35*	.24*	.23*
(6) Psychomotor retardation	1.8 (1.1)	1–5						1.00	.51*	.26*	.40*	.18*	.33*	.31*	.46*	.31*	.19*	.13*
(7) Restlessness	1.7 (1.1)	1–5							1.00	.25*	.25*	.25*	.23*	.31*	.37*	.33*	.10	.18*
(8) Insomnia	2.4 (1.3)	1–5								1.00	.18*	.32*	.22*	.28*	.39*	.27*	.15*	.17*
(9) Hypersomnia	1.8 (1.1)	1–5									1.00	.13*	.29*	.29*	.37*	.29*	.18*	.18*
(10) Appetite loss	1.6 (1.1)	1–5										1.00	.01	.32*	.31*	.26*	.16*	.07
(11) Appetite gain	2.1 (1.2)	1–5											1.00	.20*	.24*	.21*	.16*	.20*
(12) Harm self	1.9 (1.3)	1–5												1.00	.47*	.52*	.25*	.33*
(13) Poor concentration	2.5 (1.3)	1–5													1.00	.61*	.21*	.38*
(14) Worthlessness	2.3 (1.3)	1–5														1.00	.26*	.39*
(15) Lovableness	0 (1)	−1.5–3															1.00	.00
(16) Success	0 (1)	−1.6–2.3																1.00
